# Prognostic value of the triglyceride-glucose index for continuous ambulatory peritoneal dialysis patients

**DOI:** 10.1186/s12882-025-04305-x

**Published:** 2025-07-07

**Authors:** Sheng Wan, Hong Zhu, Da He, Yanglin Hu, Zengsi Wang, Yanmin Zhang

**Affiliations:** 1 Department of Nephrology, Wuhan No.1 Hospital, No. 215 of Zhongshan Avenue, Wuhan, 430030 China; 2 Department of Neuropsychology, Zhongnan Hospital of Wuhan University, Wuhan, 430071 China

**Keywords:** Triglyceride-glucose index, Continuous ambulatory peritoneal dialysis, Mortality, End-stage kidney disease, Peritonitis

## Abstract

**Objective:**

We are committed to demonstrating that the initial triglyceride-glucose (TyG) index possesses prognostic importance for patients undergoing continuous ambulatory peritoneal dialysis (CAPD).

**Methods:**

354 patients who utilized continuous CAPD at Wuhan No.1 Hospital were recruited. The participants were classified into three unique groups according to the tertiles of the TyG index. The main endpoints were overall and cardiovascular (CVD) mortality, whereas the secondary outcomes were peritonitis, technical failure, and early-onset peritonitis. The association between the TyG index and outcomes utilizing Cox proportional hazard and restricted cubic spline analysis.

**Results:**

Over a median follow-up of 72 months, 92 patients (26.0%) and 60 patients (16.9%) succumbed to all-cause and CVD mortality, respectively. The three groups exhibited significant differences for all-cause death (16.8% in tertile 1, 24.8% in tertile 2, and 36.4% in tertile 3). After full adjustment, patients with the highest TyG index demonstrated a significantly increased all-cause death relative to those in the lowest tertile (hazard ratio, HR, 2.31, 95% confidence interval, 95%CI 1.18–4.56, *P* = 0.015). Similarly, each unit increase in the TyG index was associated with a 1.32-fold elevated risk of all-cause death (HR 1.32, 95% CI 1.06–1.87, *P* = 0.031). Restricted cubic spline analysis indicated a relationship between the TyG index and all-cause mortality (P for nonlinearity > 0.05). Furthermore, the same findings were also seen with CVD mortality and secondary outcomes.

**Conclusion:**

The TyG index may function as a meaningful and reliable prognostic indicator in CAPD patients, suggesting its potential importance in improving risk stratification in clinical environments.

## Introduction

Continuous ambulatory peritoneal dialysis (CAPD) has emerged as a prevalent renal replacement therapy (RRT) for those with end-stage kidney disease (ESKD), owing to its substantial advantages in preserving residual kidney function, enhancing treatment accessibility, and lowering healthcare expenses [1, 2]. Nevertheless, the mortality rate among PD patients has remained unacceptably elevated in recent decades, with cardiovascular disease (CVD) being the primary cause of death [3, 4]. Consequently, it is essential for doctors to prioritize the pursuit of user-friendly technologies for the prompt detection of those at risk of mortality, which may enhance monitoring methods and subsequently improve the prognosis of PD patients.

The triglyceride glucose (TyG) index, which combines fasting blood glucose (FPG) and triglyceride levels (TG), was initially introduced by Unger G et al. in 2013 [5]. It has been recognized as a valuable marker of insulin resistance, a significant factor in the progression of renal illness and other conditions [6, 7]. Multiple studies have established a significant association between the TyG index and adverse clinical outcomes in kidney illnesses, such as acute kidney injury and chronic kidney disease [8, 9]. A recent report also found a tight correlation between the TyG index and the initial episode of peritonitis in PD patients [10]. Nonetheless, to the best of our knowledge, the prognostic significance of the TyG index for CAPD patients remains completely unexamined. This study assessed the relationship between the TyG index and clinical outcomes utilizing real-world data from CAPD patients at our institution.

## Materials and methods

### Patients and study design

**Fig. 1:**
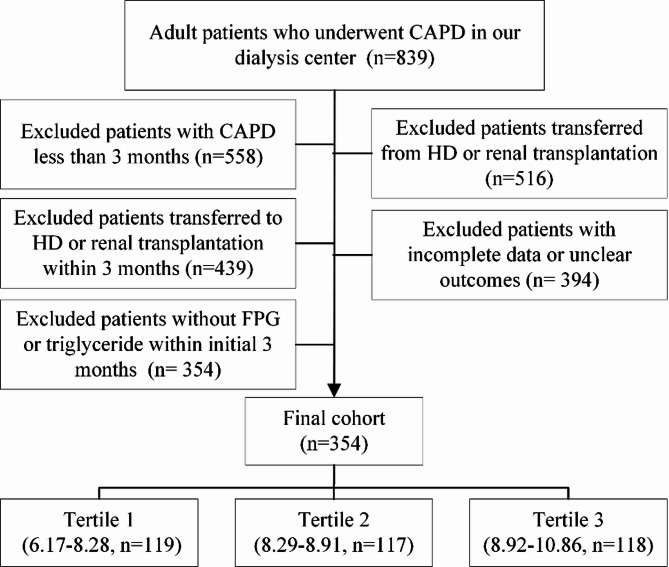
The flow chart of this study

Between 1 March 2010 and 31 December 2022, participants in this single-site retrospectively cohort study utilized CAPD as their initial renal replacement therapy and had a catheter implanted at the peritoneal dialysis center of Wuhan No. 1 Hospital. Our dialysis clinic is among one of the largest in central China, accommodating approximately 400 PD patients together with more nearly 700 HD patients receiving treatment at our facility daily. This study encompassed the subsequent patient categories, as previously described [11]: (1) Adult patients who just began peritoneal dialysis therapy; (2) Patients who have been undergoing PD treatment at our institution for over three months. We further excluded patients who were transitioned from hemodialysis or kidney transplantation, referred to other peritoneal dialysis clinics, or commenced peritoneal dialysis therapy within three months following hemodialysis or kidney transplantation. Additionally, we removed patients lacking measurements of FPG or triglyceride levels, or those with inadequate essential baseline information (missing rate > 20%) (Fig. 1). The Ethics Committee of Wuhan No. 1 Hospital accepted this study without requiring informed permission from the participants.

### Characteristics of patients

Baseline characteristics, including demographic variables, marital and educational status, primary causes of ESKD, complications, medication usage, laboratory results, and PD-related indicators, were extracted from our PD database and medical records. Laboratory data were acquired from our hospital’s biochemical laboratory using standard methods, with all studies conducted within three months after initiating PD medication. Cardiovascular disease is characterized by congestive heart failure, ischemic heart disease, cerebrovascular disease, and peripheral vascular disease. Residual kidney function, which was assessed as the mean of urea and creatinine clearance (Ccr) from the 24-h urine collection, total weekly Kt/V, and the fourth-hour dialysate to plasma creatinine ratio were computed and documented using PD Adequest Software (Baxter Healthcare Co., Ltd.).

### Definition of the TyG index

The TyG index was computed as Ln (triglycerides(mg/dL) × FPG(mg/dL)/2), as previously published [5]. Triglycerides and glucose levels were assessed using an enzymatic colorimetric assay within 1–3 months following the commencement of PD medication. All CAPD patients were classified according to the TyG tertile cutoff values (tertile 1, < 8.29; tertile 2, 8.29–8.91; tertile 3, > 8.91).

### Outcomes

The main outcomes were all-cause death and CVD mortality. CVD mortality was characterized as death resulting from myocardial infarction, heart failure, cerebrovascular accident, or peripheral vascular accident. The medical data validated the diagnosis of in-hospital death. In cases of fatalities outside the hospital, the cause of death was determined independently by two specialists at our dialysis facility, who evaluated the nursing staff’s descriptions as well as the patient’s medical record. All patients were observed throughout their therapy, including for fatal outcomes, peritoneal dialysis, and kidney transplantation. The secondary outcomes were peritonitis, technical failure, and early-onset peritonitis. Peritonitis was characterized by the fulfillment of at least two of the following criteria: [1] turbid peritoneal dialysis effluent; [2] a white blood cell counts in peritoneal dialysis effluent over 100/mm3 with 50% polymorphonuclear leukocytes; and [3] a positive culture from peritoneal dialysis effluent. Early-onset peritonitis is defined as peritonitis developing within six months of commencing PD treatment. Technique failure was characterized by mortality and the necessity for permanent hemodialysis transfer. Each patient was monitored at least once monthly utilizing our center’s medical information database or outpatient dialysis clinics. Survival time was defined as the duration from enrollment to death or administrative censoring, which included kidney transplantation, transfer to hemodialysis or other dialysis facilities, loss to follow-up, or the conclusion of the research period (October 1, 2024).

### Statistics

All computations were conducted using the statistical tool R (version 4.1.0). The baseline features of TyG tertile groups were compared using one-way ANOVA for continuous variables and the Pearson chi-square test for categorical variables. Kaplan-Meier survival analysis was used to assess the incidence rate of endpoints in groups classified by various TyG index values, with differences examined using log-rank testing. We also used limited cubic spline regression to investigate the nonlinear connection between the baseline TyG score and both overall and cardiovascular disease mortality. Multivariate Cox regression analysis were used to determine the relationship between the TyG index and the risk of major outcomes. The logistic regression, odds ratios (ORs), and 95% confidence intervals (CIs) were used to assess the TyG index’s impact on secondary outcomes. The TyG index was entered into models as both a continuous variable and a categorical variable (the tertile of the TyG index). P values for trends across the tertiles of the TyG index were assessed using the median value within each tertile as a predictor. Clinically relevant and prognosis-associated variables were incorporated into the multivariate model: model 1: unadjusted; model 2: adjusted for body mass index, marital status, educational status, and etiology of ESRD; model 3: adjusted for model 2 plus comorbidities, treatments, dialysis dose, and laboratory results (excluding triglyceride and glucose). A p-value of less than 0.05 was employed to signify a statistically significant difference in either direction.

## Results

### Characteristics of patients

In the current retrospective study, a total of 354 patients were gathered, including 183 males (51.7%) and 171 women (48.3%), with a mean age of 52.2 years (range: 18–83). A minority of patients (72, 20.3%) attained a college education or above, whereas the majority (2329, 92.9%) were married at the commencement of their initial PD therapy. In our CAPD patients, hypertension was the predominant comorbidity (332, 93.8%), after diabetes (99, 28.0%), and 30 individuals (8.5%) had a history of CVD. Chronic glomerulonephritis (169, 47.7%) was the predominant cause of ESKD, immediately following diabetes (80, 22.6%), hypertension (63, 17.8%), and other or unidentified causes (42, 11.8%). Table 1 presents the information of all patients, categorized into three groups based on tertiles of the TyG index (T1: 6.17–8.28; T2: 8.29–8.91; T3: 8.92–10.86). Patients in the highest tertile of the TyG index exhibited a greater prevalence of diabetes and elevated levels of white blood cells, blood urea nitrogen, creatinine, total cholesterol, triglycerides, LDL-C, and glucose, alongside reduced levels of hemoglobin and HDL-C, compared to those in the lowest tertiles (all *P* < 0.05, Table 1). Moreover, relative to the lowest tertile of TyG, patients in the highest tertile exhibited an increased incidence of overall death, cardiovascular mortality, peritonitis, technical failure, and early-onset peritonitis (all *P* < 0.05, Table 1).

**Table 1:** Baseline characteristics of all patients in this study

Characteristics	T1 (6.17–8.28)	T2 (8.29–8.91)	T3 (8.92–10.86)	*P* value
N	119	117	118	-
Age, years old	50.5 ± 14.1	51.7 ± 12.7	54.4 ± 13.4	0.069
Gender, male, n (%)	71 (59.7)	55 (47.0)	57 (48.3)	0.105
Body mass index, kg/m^2^	22.9 ± 3.7	22.4 ± 3.5	23.2 ± 3.5	0.224
Duration of dialysis, months	21.0 (4.0, 54.0)	23.0 (3.0, 54.0)	23.0 (3.0, 59.3)	0.987
CCI, points	4.0 (2.0, 5.0)	4.0 (2.0, 5.0)	5.0 (2.8, 6.0)	0.581
Marital status, n (%)				0.064
Unmarried	14 (11.8)	6 (5.1)	5 (4.2)	
Married	105 (88.2)	111 (94.9)	113 (95.8)	
Educational status, n (%)				0.377
Illiterate/primary	15 (12.6)	17 (14.5)	16 (13.6)	
Middle school	44 (37.0)	34 (29.1)	47 (39.8)	
Secondary school	32 (26.9)	45 (38.5)	32 (27.1)	
University or above	28 (23.5)	21 (17.9)	23 (19.5)	
Cause of ESRD, n (%)				0.154
Chronic glomerulonephritis	56 (47.1)	69 (59.0)	44 (37.3)	
Diabetic nephropathy	27 (22.7)	15 (12.8)	38 (32.2)	
Hypertensive nephropathy	18 (15.1)	22 (18.8)	23 (19.5)	
Others or unknown	18 (15.1)	11 (9.4)	13 (11.0)	
Comorbidities, n (%)				
Cardiovascular disease	14 (11.8)	6 (5.1)	10 (8.5)	0.191
Hypertension	110 (92.4)	111 (94.9)	111 (94.1)	0.792
Diabetes	33 (27.7)	21 (17.9)	45 (38.1)	0.002
Treatments, n (%)				
ACEI/ARB	80 (67.2)	73 (62.4)	67 (56.8)	0.255
βblockers	57 (47.9)	68 (58.1)	66 (55.9)	0.252
CCB	103 (86.6)	97 (82.9)	104 (88.1)	0.513
Diuretics	6 (5.0)	5 (4.3)	9 (7.6)	0.549
Statins/fibrates	12 (10.1)	18 (15.4)	16 (13.6)	0.451
Dialysis dose				
Weekly total Ccr	65.2 ± 15.7	65.0 ± 14.7	60.6 ± 14.2	0.285
Weekly renal Ccr	20.5 ± 6.2	20.0 ± 7.6	17.3 ± 6.9	0.616
Weekly total Kt/Vurea	1.7 ± 0.6	1.9 ± 0.7	2.2 ± 0.8	0.225
Weekly renal Kt/Vurea	0.6 ± 0.2	0.4 ± 0.1	0.3 ± 0.1	0.332
PET at baseline	0.7 ± 0.1	0.6 ± 0.1	0.7 ± 0.1	0.151
Laboratory results				
White blood cells, × 10^9^/L	6.3 ± 2.0	7.0 ± 2.5	8.0 ± 2.7	< 0.001
Hemoglobin, g/L	103.9 ± 23.0	112.0 ± 20.5	109.4 ± 20.5	0.013
Platelets, × 10^9^/L	203.1 ± 51.5	220.9 ± 56.4	218.5 ± 51.2	0.103
Albumin, g/L	36.8 ± 3.7	36.9 ± 3.8	36.5 ± 3.6	0.738
Blood urea nitrogen, µmol/L	18.5 ± 5.1	19.4 ± 4.9	21.0 ± 5.7	0.001
Serum creatinine, µmol/L	943.3 ± 128.4	956.9 ± 119.6	1076.2 ± 130.7	0.002
Total cholesterol, mmol/L	3.9 ± 1.2	4.6 ± 0.7	4.8 ± 0.9	< 0.001
Triglyceride, mmol/L	1.0 ± 0.3	1.8 ± 0.5	4.0 ± 1.2	< 0.001
HDL-C, mmol/L	1.3 ± 0.3	1.2 ± 0.4	1.1 ± 0.3	0.018
LDL-C, mmol/L	1.8 ± 0.7	2.0 ± 0.7	2.2 ± 0.8	< 0.001
Glucose, mmol/L	5.5 ± 1.1	6.3 ± 1.4	8.7 ± 2.9	< 0.001
TyG index	7.9 ± 0.6	8.6 ± 0.2	9.4 ± 0.6	< 0.001
Calcium, mmol/L	2.3 ± 0.2	2.3 ± 0.3	2.4 ± 0.2	0.599
Phosphorus, mmol/L	1.8 ± 0.5	1.8 ± 0.5	1.7 ± 0.4	0.608
iPTH, pg/mL	342.0 (166.5, 701.3)	268.0 (89.6, 602.5)	357.0 (135.9, 641.6)	0.523
hsCRP, mg/L	3.1 (1.1, 6.3)	3.4 (1.5, 10.8)	3.8 (2.9, 10.2)	0.547
Clinical outcomes, n (%)				
Overall death	20 (16.8)	29 (24.8)	43 (36.4)	0.002
CVD death	11 (9.2)	16 (13.7)	33 (28.0)	< 0.001
Survival months	60.0 (40.0, 95.0)	60.0 (39.5, 95.0)	63.5 (38.8, 93.8)	0.941
Kidney transplant	7 (5.9)	6 (5.1)	5 (4.2)	0.847
Peritonitis	38 (31.9)	51 (43.6)	68 (57.6)	< 0.001
Early peritonitis	5 (4.2)	9 (7.7)	32 (27.1)	< 0.001
Total number of peritonitis^1^	1.7 ± 0.4	1.5 ± 0.4	1.8 ± 0.5	0.256
Technical failure	20 (16.8)	21 (17.9)	40 (33.9)	0.003

^1^Total number of peritonitis, excluded patients without peritonitis

CCI, Charlson comorbidity index, ESRD, end-stage renal disease, ACEI/ARB, angiotensin converting enzyme inhibitors/angiotensin receptor blocker, CCB, calcium calcium blockers, Ccr, Creatinine clearance, PET, peritoneal equilibration test, HDL-C, high-density lipoprotein cholesterol, LDL-C, low-density lipoprotein cholesterol, TyG index, triglyceride-to-glucose index, iPTH, intact parathyroid hormone, CRP, c-reactive protein, CVD, cardiovascular disease

### The predictive capacity of the TyG index for the key outcomes

**Fig. 2:**
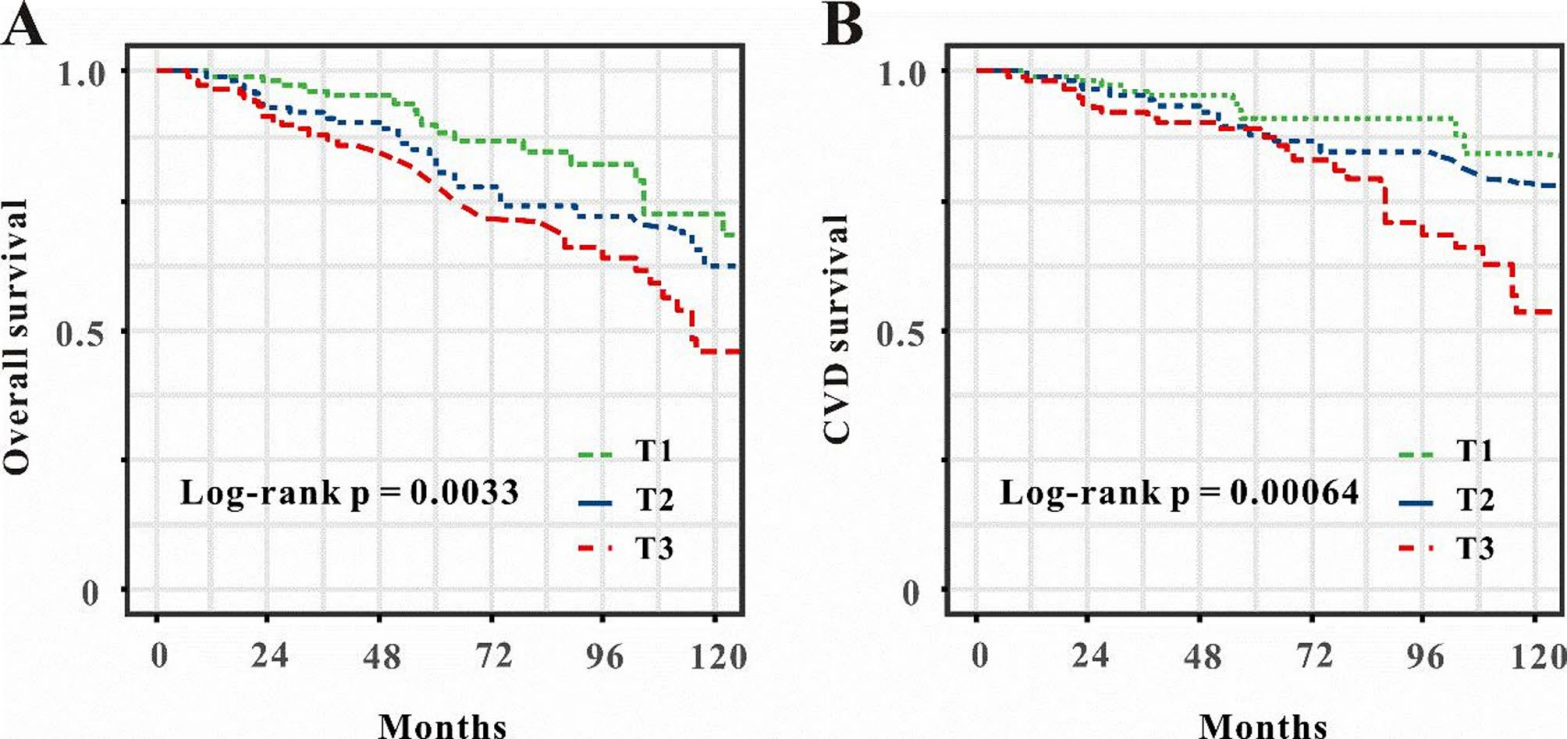
Kaplan-Meier survival analysis curves of the tertile of TyG index for overall mortality (**A**), and CVD mortality (**B**). TyG index, triglyceride-glucose index, CVD, cardiovascular disease

The Kaplan-Meier survival analysis curves were utilized to examine the occurrence of primary outcomes among groups, according to the tertiles of the TyG index illustrated in Fig. 2A-B. Patients with the highest TyG score demonstrated the greatest risk of overall death and CVD mortality. Table 2 delineates the findings of the multivariable Cox proportional hazards regression analysis. The results indicated that the TyG index was a significant risk factor for overall mortality in model 3 (HR: 1.32, 95%CI 1.06–1.87, *P* = 0.031) when treated as a continuous variable (Table 2). In the context of the TyG index as a nominal variable, patients in the highest tertile exhibited a substantially elevated risk of all-cause mortality compared to those in the lowest tertile, after adjustment for full model (HR: 2.31, 95% CI: 1.18–4.56, *P* = 0.015). Comparable outcomes were noted in the multivariate Cox proportional hazards analysis of the TyG index and cardiovascular disease mortality (Table 2).

**Table 2:** The association between the TyG index and overall and CVD mortality

Exposure	Model 1		Model 2		Model 3	
	HR (95%CI)	*P*	HR (95%CI)	*P*	HR (95%CI)	*P*
Overall mortality						
TyG as continuous	1.38 (1.06–1.79)	0.017	1.40 (1.05–1.68)	0.021	1.32 (1.06–1.87)	0.031
T1	Ref.		Ref.		Ref.	
T2	1.63 (0.91–2.92)	0.102	1.63 (0.89–2.96)	0.112	1.47 (0.77–2.81)	0.246
T3	2.46 (1.43–4.24)	0.001	2.33 (1.14–3.48)	0.015	2.31 (1.18–4.56)	0.015
P for trend		0.004		0.042		0.015
CVD mortality						
TyG as continuous	1.53 (1.11–2.13)	0.010	1.37 (1.06–1.89)	0.026	1.49 (1.08–2.06)	0.017
T1	Ref.		Ref.		Ref.	
T2	1.55 (0.71–3.36)	0.269	1.64 (0.74–3.62)	0.220	2.06 (0.91–4.28)	0.059
T3	3.24 (1.62–6.45)	< 0.001	2.79 (1.38–5.64)	0.004	4.48 (1.51–13.56)	0.008
P for trend		0.001		0.011		0.006

TyG, triglyceride-glucose index, CVD, cardiovascular disease, HR, hazard ratio, 95%CI, 95% confidence index, Model 1 was unadjusted, model 2 adjusted for age, gender, body mass index, duration of dialysis, marital status, educational status, and cause of ESRD. Model 3 adjusted for model 2 plus Charlson comorbidity index, comorbidities, treatments, dialysis dose, and laboratory results (except for triglyceride and glucose)

**Fig. 3:**
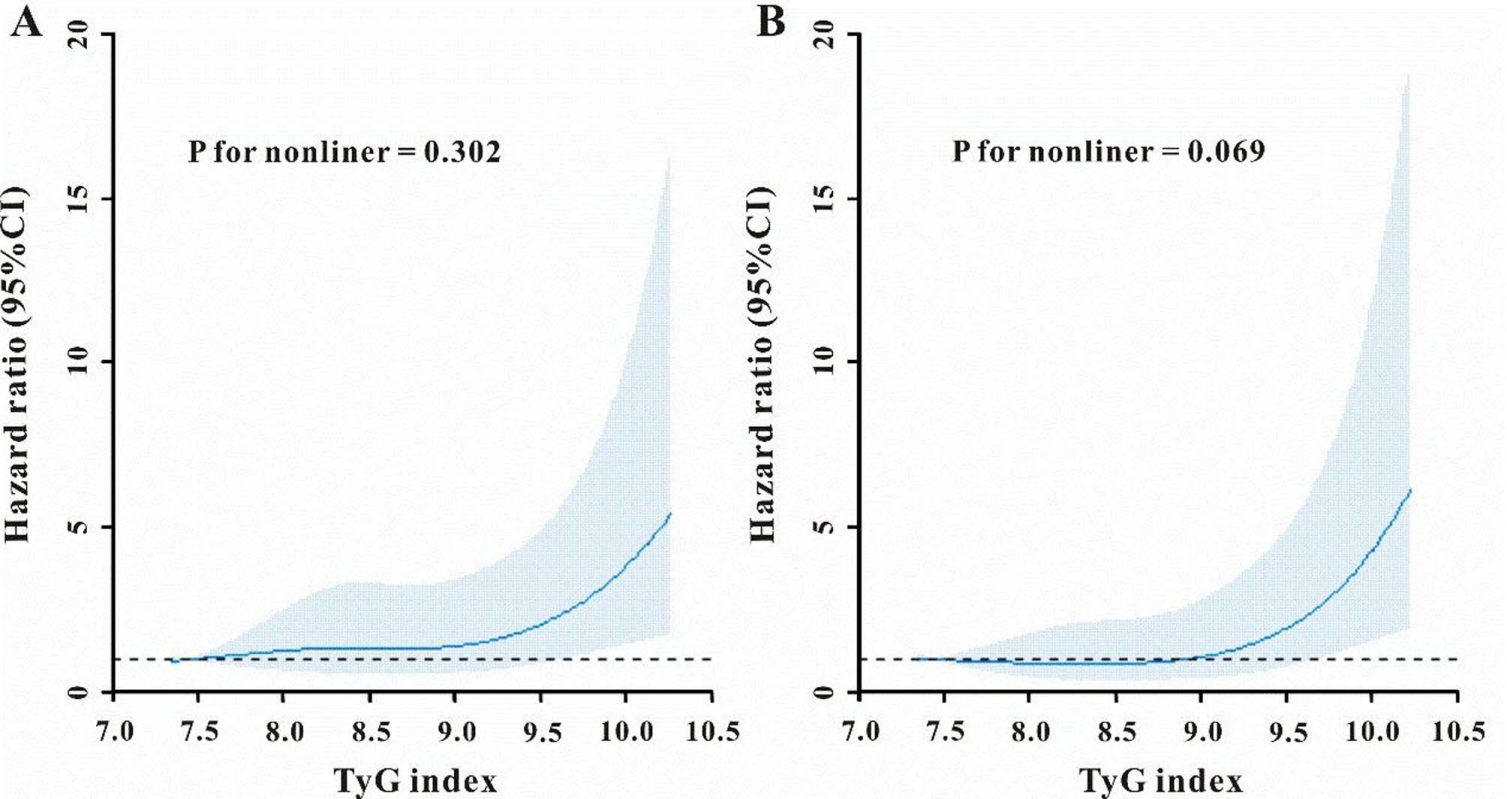
Restricted cubic spline curve for all-cause mortality (**A**), and CVD mortality(**B**). CI, confidence interval, TyG index, triglyceride-glucose index, CVD, cardiovascular disease

The restricted cubic splines regression model was utilized to illustrate the risk of death, revealing a linear increase in all-cause mortality, and CVD mortality corresponding to the rising TyG index (P for non-linearity were 0.302 and 0.069, respectively, Fig. 3A-B).

### The predictive capacity of the TyG index for the secondary outcomes

Aside from the major outcomes, we then attempted to assess the predictive capacity of the TyG index for forecasting secondary endpoints. Table 3 presents the findings of the multivariable logistic regression analysis. The results demonstrated that the TyG index was a significant risk factor for peritonitis in model 3 (OR: 1.78, 95% CI: 1.25–2.53, *P* = 0.001) when treated as a continuous variable. When the TyG index was treated as a nominal variable, patients in the highest tertile of the TyG index exhibited a substantially elevated risk of peritonitis compared to those in the lowest tertile, after model adjustment (OR: 3.32, 95% CI: 1.81–6.11, *P* < 0.001). Comparable outcomes were also noted in the multivariable logistic regression analysis of the TyG index for the prediction of technical failure and early-onset peritonitis (Table 3).

**Table 3:** The association between the TyG index and other clinical outcomes

Exposure	Model 1		Model 2		Model 3	
	OR (95%CI)	*P*	OR (95%CI)	*P*	OR (95%CI)	*P*
Peritonitis						
TyG as continuous	1.82 (1.36–2.43)	< 0.001	1.79 (1.34–2.40)	< 0.001	1.78 (1.25–2.53)	0.001
T1	Ref.		Ref.		Ref.	
T2	1.65 (0.97–2.80)	0.066	1.61 (0.94–2.76)	0.085	1.63 (0.90–2.95)	0.105
T3	2.90 (1.71–4.93)	< 0.001	2.84 (1.66–4.89)	< 0.001	3.32 (1.81–6.11)	< 0.001
P for trend		< 0.001		0.001		0.006
Technical failure						
TyG as continuous	1.60 (1.16–2.31)	0.001	1.60 (1.15–2.23)	0.004	1.66 *1.11–2.49)	0.013
T1	Ref.		Ref.		Ref.	
T2	1.08 (0.55–2.12)	0.817	1.09 (0.55–2.17)	0.807	1.12 (0.51–2.45)	0.774
T3	2.54 (1.38–4.69)	0.003	2.58 (1.38–4.85)	0.003	2.14 (1.14–4.50)	0.026
P for trend	0.003			0.005		0.016
Early peritonitis						
TyG as continuous	2.02 (1.34–3.04)	0.001	1.91 (1.27–2.88)	0.002	1.99 (1.22–3.25)	0.006
T1	Ref.		Ref.		Ref.	
T2	0.85 (0.68–3.68)	0.291	0.94 (0.57–5.69)	0.292	0.75 (0.31–2.15)	0.483
T3	3.55 (2.06–9.04)	< 0l001	3.31 (1.91–9.69)	< 0.001	3.67 (2.09–14.19)	< 0.001
P for trend		< 0.001		< 0.001		< 0.001

TyG, triglyceride-glucose index, ARF, acute respiratory failure, OR, odds ratio, 95%CI, 95% confidence index, Model 1 was unadjusted, model 2 adjusted for age, gender, body mass index, duration of dialysis, marital status, educational status, and cause of ESRD. Model 3 adjusted for model 2 plus Charlson comorbidity index, comorbidities, treatments, dialysis dose, and laboratory results (except for triglyceride and glucose)

### Analysis of subgroups

**Fig. 4:**
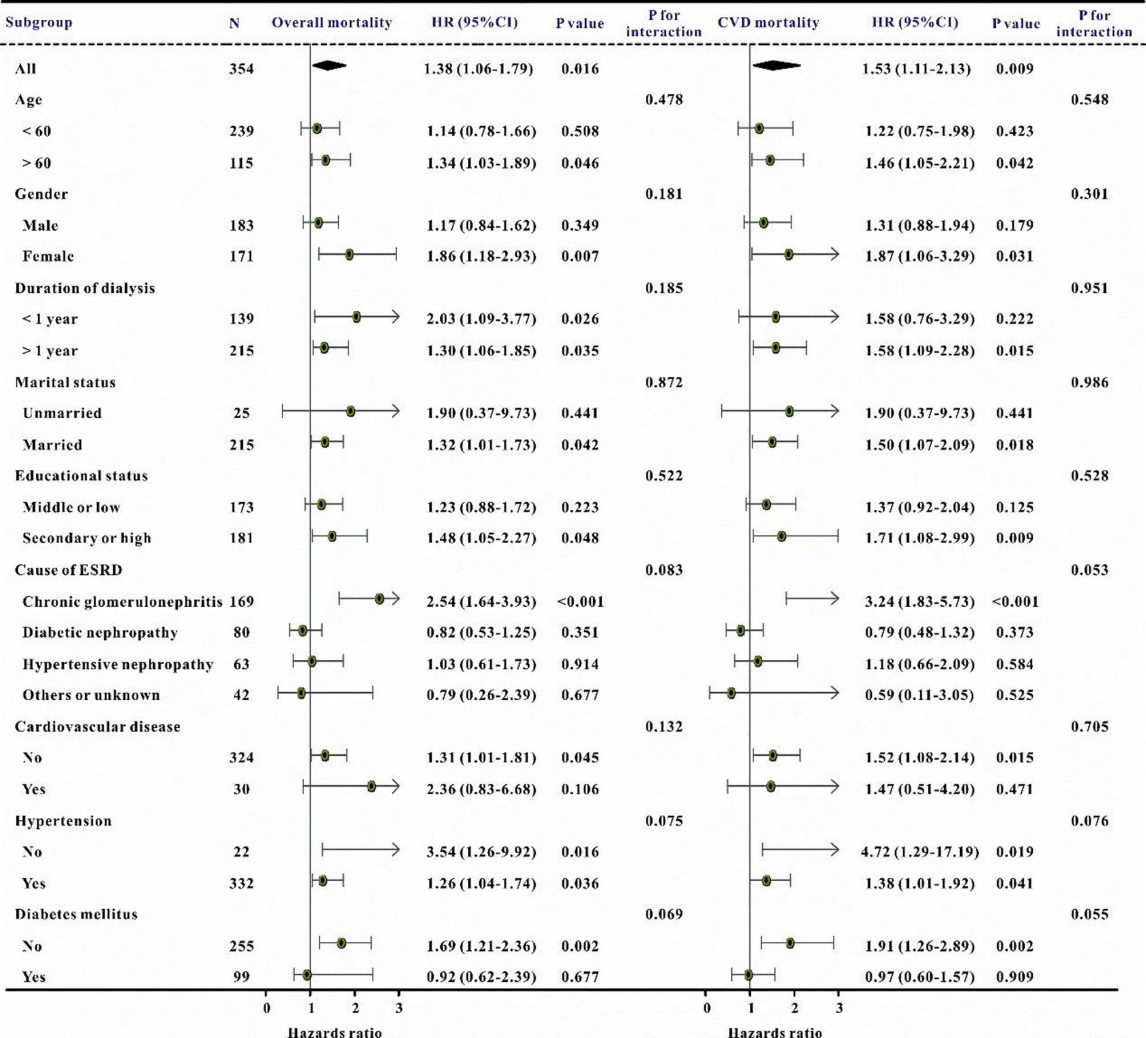
The forest plot revealed the results of subgroup analysis for all-cause mortality, and CVD mortality, respectively, based on the TyG index. HR, hazard ratio, CI, confidence interval, TyG index, triglyceride-glucose index, CVD, cardiovascular disease

Additionally, subgroup analyses were performed to confirm the relationship between the TyG index and main outcomes, categorized by age, gender, length of dialysis, marital status, educational attainment, etiology of ESKD, cardiovascular disease, hypertension, and diabetes mellitus. The TyG score was substantially correlated with an increased risk of overall mortality and cardiovascular death in the majority of subgroups (Fig. 4).

## Discussion

This retrospective study examined the original TyG index as a prognostic indicator for first-time CAPD patients, utilizing real-world data from 354 individuals at our dialysis clinic. Our findings indicate that the TyG index had sufficient predictive capability for all-cause and CVD mortality. Moreover, a raised TyG index correlated with a heightened risk of peritonitis, technical failure, and early-onset peritonitis. This association persisted strongly, even after accounting for potential affecting factors. Consequently, the data indicated that early TyG may serve as a valid and declaring anticipatory predictor for CAPD patients.

Despite significant advancements in knowledge and facilities in recent decades, the prognosis for individuals with CAPD has continuously been dismal, with CVD identified as the primary cause of mortality, accounting for 48-70% in previous studies [12, 13]. Song et al. performed a multi-center retrospective study with 586 CAPD patients and found an overall mortality rate of 8.7%, with CVD responsible for about 60.0% (51/85) of all-cause fatalities [14]. A recent study including 188 CAPD patients revealed an overall mortality rate of 39.9% (75/188) over a median follow-up period of 60 months [14]. In contrast to the prior study, our dialysis facility included 354 CAPD patients and found a total mortality rate of 26.0%, with about 65.2% (60/92) of dead patients succumbing to cardiovascular disease, aligning with past studies. Consequently, a dependable and creative biomarker or predictive model for the mortality of CAPD patients may be established to enable physicians to stratify risk, thus facilitating the timely and effective administration of treatments to enhance patient prognoses and reduce healthcare costs.

The TyG index, which integrates fasting blood glucose and triglyceride levels, can be readily calculated in clinical practice cost-effectively using standard blood biochemical tests. This index has been employed by numerous researchers to predict various clinical outcomes across several diseases, including kidney diseases, which piqued our significant interest. Jin et al. sought to evaluate the predictive capacity of the TyG index for acute kidney damage in ICU patients, and the findings demonstrated a correlation between the TyG index and the risk of acute renal injury in this population [15]. Ye et al. investigated the correlation between the TyG index and the prognosis of patients with chronic kidney disease (CKD) admitted to the ICU, revealing that the TyG index serves as a predictor of one-year mortality and in-hospital mortality in CKD patients [9]. Fritz et al. conducted a prospective, community-based cohort analysis involving 176,420 Austrian individuals, revealing that the TyG index is related to the risk of ESKD and mediates about half of the overall connection between body mass index and ESKD in the general population [16]. Comparable findings have also been reported in another prior research [17, 18]. In individuals with ESKD, the TyG index significantly influenced their prognosis. Karabulut et al. included 522 non-diabetic patients having renal transplantation and established that the TyG index serves as an independent predictor of adverse outcomes [19]. Engin et al. conducted a retrospective analysis including 189 diabetic hemodialysis patients who had arteriovenous fistula (AVF) surgery, concluding that the TyG index was positively correlated with primary AVF failure [20]. Furthermore, a recent study indicated that the baseline serum TyG index serves as an independent risk factor for the initial episode of peritonitis in patients undergoing peritoneal dialysis [10]. Nonetheless, the significance of the TyG index concerning unfavorable outcomes in CAPD patients remains predominantly unclear. The current study’s findings corroborate that a high TyG index is connected with an increased risk of all-cause mortality, cardiovascular disease mortality, as well as a heightened risk of peritonitis, technical failure, and early-onset peritonitis. This research enhances the significance of the TyG index in forecasting outcomes for CAPD patients.

The precise processes governing the association between the TyG index and clinical outcomes in CAPD patients are currently unidentified. Insulin resistance (IR) may be the underlying mechanism. IR denotes the diminished ability of insulin to promote the uptake and use of glucose, indicating a failure in glucose metabolism. Growing data indicates that insulin resistance can result in cardiovascular diseases in both diabetic and non-diabetic persons [21]. Patients with insulin resistance are susceptible to several metabolic diseases, including hyperglycemia, dyslipidemia, and hypertension, all of which are intricately linked to negative consequences [22, 23]. Moreover, heightened oxidative stress seems to be a detrimental component contributing to insulin resistance, and it has also been noted to significantly impact individuals undergoing CAPD [24–26]. These pathophysiological changes jointly promote the initiation and advancement, resulting in negative clinical consequences. The specific processes require additional investigation in further research.

This retrospective study has some limitations. Initially, we were limited to describing the events and were unable to establish causal inferences. Secondly, it was a retrospective, single-center research with a somewhat limited sample size. Thirdly, we exclusively recorded the first TyG parameter readings; these values may fluctuate during subsequent follow-ups at our institution, influencing the patient’s clinical outcomes. Furthermore, we could not account for the possible influences of variables such as medication and exposure to glucose-based peritoneal dialysis solutions, nor could we exclude all potential confounding factors. Ultimately, due to the study’s sample being confined to CAPD patients, the applicability of the findings to hemodialysis patients or those with ESKD not undergoing dialysis necessitates validation in future research.

## Conclusions

In conclusion, we originally showed that the TyG index, a straightforward and cost-effective metric, may function as an effective risk classification instrument for CAPD patients. Moreover, considering that the TyG index is a practical and cost-effective laboratory indicator, it may possess greater clinical relevance and potential for implementation in clinical settings.

## Data Availability

Data is provided within the manuscript or supplementary information files.

## Declarations

### Patient and public involvement

Patients and/or the public were not involved in the design, conduct, reporting, or dissemination plans of this research.

### Provenance and peer review

Not commissioned; externally peer-reviewed.
